# COVID-19 Pandemic and Infant Neurodevelopmental Impairment: A Systematic Review and Meta-analysis

**DOI:** 10.1001/jamanetworkopen.2022.38941

**Published:** 2022-10-28

**Authors:** Kamran Hessami, Amir Hossein Norooznezhad, Sonia Monteiro, Enrico R. Barrozo, Abolfazl Shirdel Abdolmaleki, Sara E. Arian, Nikan Zargarzadeh, Lara S. Shekerdemian, Kjersti M. Aagaard, Alireza A. Shamshirsaz

**Affiliations:** 1 Maternal Fetal Care Center, Boston Children’s Hospital, Harvard Medical School, Boston, Massachusetts; 2 Medical Biology Research Center, Health Technology Institute, Kermanshah University of Medical Sciences, Kermanshah, Iran; 3 Meyer Center for Developmental Pediatrics and Autism, Department of Pediatrics, Texas Children’s Hospital, Baylor College of Medicine, Houston; 4 Division of Maternal Fetal Medicine, Texas Children’s Hospital, Baylor College of Medicine, Houston; 5 Maternal Fetal Medicine Research Center, Shiraz University of Medical Sciences, Shiraz, Iran; 6 Department of Obstetrics and Gynecology, Baylor College of Medicine, Houston, Texas; 7 School of Medicine, Tehran University of Medical Sciences, Tehran, Iran; 8 Division of Critical Care, Department of Pediatrics, Texas Children’s Hospital, Baylor College of Medicine, Houston

## Abstract

**Importance:**

Primary studies proposed that aberrant maternal antiviral immunity and/or giving birth in quarantine, such as during the ongoing COVID-19 pandemic, may be associated with the risk of neurodevelopmental impairment (NDI) in offspring.

**Objectives:**

To evaluate the associations of birth and being raised during the COVID-19 pandemic with risk of NDI among infants and to assess the association of gestational exposure to SARS-CoV-2 with risk of NDI.

**Data Sources:**

PubMed, Web of Science, Scopus, Embase, and preprint servers were systematically searched from inception to March 25, 2022.

**Study Selection:**

Studies evaluating the neurodevelopment of infants born during the SARS-CoV-2 pandemic were included in this systematic review and meta-analysis. Studies using Ages and Stages Questionnaires, Third Edition (ASQ-3), were used for quantitative meta-analysis.

**Data Extraction and Synthesis:**

Following the Preferred Reporting Items for Systematic Reviews and Meta-analyses, a random-effects model meta-analysis was used to pool the proportion and odds ratios (ORs) of overall NDI, as well as each developmental domain on ASQ-3 with the corresponding 95% CI.

**Main Outcomes and Measures:**

The primary outcome was the risk of overall NDI among infants screened during the pandemic vs prepandemic. The secondary outcome was the comparison of NDI by ASQ-3 domain among infants born to women with known gestational exposure to SARS-CoV-2 vs no exposure.

**Results:**

A total of 8 studies were included, including 21 419 infants (11 438 screened in pandemic and 9981 in prepandemic period). NDI was present in 330 of 8992 infants (7%; 95% CI, 4%-10%) screened during the COVID-19 pandemic from January 2020 to January 2021. Among the pandemic cohort, the prevalence of NDI among infants with gestational exposure to SARS-CoV-2 was 77 of 691 (12%; 95% CI, 6%-18%). Compared with the prepandemic cohort (2015-2019), the pandemic cohort was more likely to have communication impairment (OR, 1.70; 95% CI, 1.37-2.11; *P* < .001), without significant differences in other ASQ-3 domains (eg, gross motor, fine motor, personal-social, and problem-solving). In contrast, maternal SARS-CoV-2 infection was not associated with significant differences in any neurodevelopment domain in offspring, except for increasing the odds of fine motor impairment (OR, 3.46; 95% CI, 1.43-8.38; *P* < .001).

**Conclusions and Relevance:**

In this systematic review and meta-analysis examining the association between COVID-19 pandemic and the risk of NDI, findings suggest that overall neurodevelopment in the first year of life was not changed by either being born or raised during the SARS-CoV-2 pandemic or by gestational exposure to SARS-CoV-2. Interestingly, the first year of life during the COVID-19 pandemic, regardless of maternal infection, was significantly associated with the risk of communication delay among the offspring.

## Key Points

**Question:**

Are neurodevelopmental outcomes during infancy changed by the COVID-19 pandemic?

**Findings:**

This meta-analysis of 8 studies including 21 419 infants found that 7% of infants who had neurodevelopmental screening during the COVID-19 pandemic were at risk of neurodevelopmental impairment, and 12% of those with gestational exposure to SARS-CoV-2 were at risk for neurodevelopmental impairment. Communication impairment was the sole neurodevelopmental domain of significantly increased risk of occurrence during the COVID-19 pandemic.

**Meaning:**

These findings suggest that overall neurodevelopment was not changed by the COVID-19 pandemic, but birth or being raised during the SARS-CoV-2 pandemic, regardless of gestational exposure, was associated with a significant risk of communication impairment among the infants.

## Teaser

This systematic review and meta-analysis evaluates whether the COVID-19 pandemic is associated with changes in neurodevelopmental outcomes during infancy.

## Introduction

COVID-19, caused by SARS-CoV-2, persists as a global health emergency.^1^ Preliminary reports showed that pregnant individuals are at a higher risk of mortality and severe morbidity due to COVID-19, and consensus from the US Centers for Disease Control and Prevention supports the disproportionate risk that pregnancy imparts.^2,3,4^ According to the results of a living systematic review and meta-analysis,^5^ 10% of pregnant women attending or admitted to hospitals for any reason, not only obstetrics-related issues, were suspected of having or had a diagnosis of COVID-19. At present, our understanding of the effects of maternal COVID-19 infection on short-term and long-term aspects of maternal-fetal health, including neurodevelopmental outcomes during infancy, remains limited.^2^

Neurodevelopmental disorders, such as autism spectrum disorder, intellectual disability, and attention-deficit/hyperactivity disorder, have heterogeneous causes associated with impaired cognition, communication, adaptive behavior, and psychomotor skills.^6^ The neurodevelopment of a fetus can be affected by different endogenous (direct fetal infection, maternal infection with vertical transmission, and neurologic abnormalities) or exogenous (maternal immune activation in the absence of vertical transmission, maternal environmental chemical or dietary factors, or marked and persistent maternal stress) factors.^7,8,9,10,11^ Maternal immune activation hypothesis proposes that inflammatory perturbations in utero can affect fetal neurodevelopment, and evidence from human epidemiological studies^8,9^ supports an association between maternal inflammation during pregnancy and offspring neurodevelopmental disorders.

Fortunately, maternal-fetal vertical transmission of SARS-CoV-2 is rare, although the impact of maternal infection on fetal and postnatal neurodevelopment is still poorly understood.^12^ Recently, a few cohort studies^13,14,15,16^ on this clinically important issue have been published with controversial results. Shuffrey et al,^15^ as one of the largest cohorts, assessed 255 infants born during the pandemic, and exposure to maternal SARS-CoV-2 infection was not associated with differences in neurodevelopmental screening scores at age 6 months, regardless of infection timing or severity. However, both infants with and without SARS-CoV-2 exposure born during that period had significantly lower scores on gross motor, fine motor, and personal-social subdomains compared with the prepandemic cohort.^15^

The net impact of the COVID-19 pandemic on potential risk of neurodevelopmental impairment (NDI) is of extreme importance. The current study aimed to determine whether (1) being born or raised during the COVID-19 pandemic and (2) gestational exposure to SARS-CoV-2 are associated with an increased risk of NDI during the first year of life using the Ages and Stages Questionnaires, Third Edition (ASQ-3), neurodevelopmental screening tool.

## Methods

This systematic review and meta-analysis was conducted according to the Preferred Reporting Items for Systematic Reviews and Meta-analyses (PRISMA) reporting guideline. The study protocol for this systematic review was registered in the PROSPERO international prospective register of systematic reviews (Registration number: CRD42022315849).

### Search Strategy

A literature search was performed by 2 independent authors (K.H. and N.Z.) using PubMed, Web of Science, and Embase from inception to March 25, 2022. The search was conducted from March 19 to March 25, 2022, with no language restriction using the following keywords: (*COVID-19* OR *Coronavirus* OR *Severe Acute Respiratory Syndrome* OR *Corona-virus* OR *2019nCoV* OR *Corona Virus* OR *COVID* OR *COVID19* OR *SARS CoV 2* OR *SARS-CoV* OR *SARS-CoV-2*) AND (*neurodevelopment* OR *neurodevelopmental* OR development disorder* OR developmental disorder* OR *intellectual disability* OR intellectual developmental disorder* OR *mental retardation* OR *global developmental delay* OR *communication disorders* OR *gross motor* OR *fine motor* OR *personal social* OR *problem solving*). The detailed search strategy is shown in eTable 1 in the Supplement. References of relevant articles were manually reviewed, and eligible studies were added to the results from the electronic literature search. Literature search and study selection were performed by 2 independent authors (N.Z. and A.N.), and discrepancies were resolved by consulting the third investigator (A.A.S.).

### Eligibility Criteria

This review included only observational studies evaluating the risk of NDI among infants who had their neurodevelopmental screening during the COVID-19 pandemic. Only studies presenting data on infants undergoing neurodevelopmental screening up to 12 months corrected age follow-up were deemed eligible for inclusion. To be eligible for quantitative synthesis, studies were included if they defined “being at risk of NDI” as a composite or domain score at least 2 SDs below the established mean of the normal population reported by the standardized neurodevelopmental assessment tool of ASQ-3. Articles designed as case reports, narrative reviews, case series, dissertations, and letters or editorials were excluded. In terms of defining comparison groups, the risk of NDI was assessed among those who had screening during the pandemic vs those who had screening before the pandemic. As a secondary analysis, the risk of NDI was also assessed among those who were born during the pandemic with documented maternal SARS-CoV-2 infection vs those born during the pandemic with no maternal SARS-CoV-2 infection.

### Study Selection

**Figure 1.**
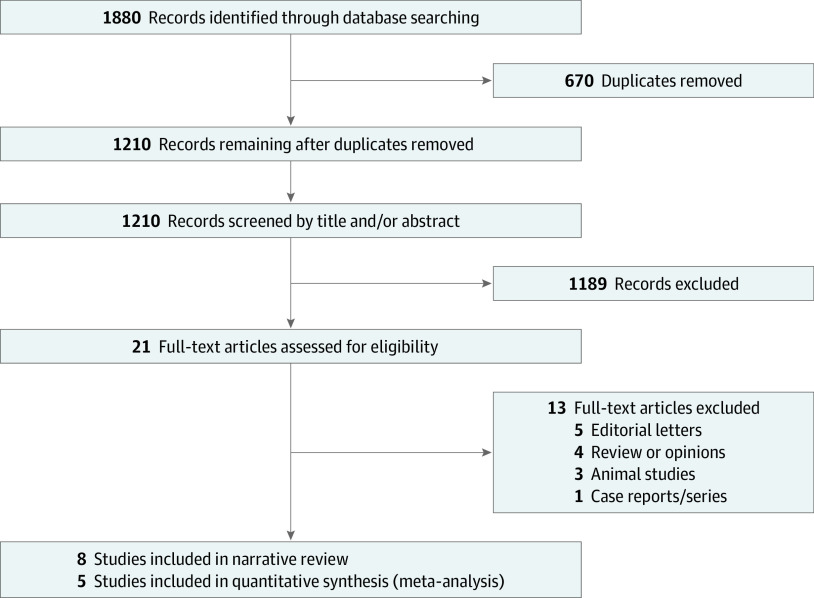
Study Flowchart

A total of 1880 articles were retrieved. Of these, 670 articles were excluded as duplicates, and the remaining 1210 studies were screened for eligibility. Screening of the title and abstract resulted in 21 potentially eligible studies. Following full-text assessment, 8 studies met the inclusion criteria defined earlier (Figure 1).

### Ages and Stages Questionnaires, Third Edition

The ASQ-3 is a parent-completed comprehensive neurodevelopmental screener with 30 items in 5 developmental domains: communication, gross motor, fine motor, problem-solving, and personal-social. Each of the 30 items describes a skill, ability, or behavior to which the parent responds yes (10 points), sometimes (5 points), or not yet (0 points).^17^ For the quantitative meta-analysis, the score calculated for each domain was categorized as normal development (above cutoff) or at risk of NDI, if the score was 2 SDs below the mean, which indicates a possible delay that requires further follow-up or referral.

### Primary and Secondary Outcomes

The primary outcome measure was the prevalence of infants (up to 12 months after birth) at risk of NDI who had their neurodevelopmental screening during the COVID-19 pandemic compared with the prevalence of infants at high risk of NDI who had their screening before the pandemic. The secondary outcome measure was to compare the risk of NDI among pandemic-born infants who had in utero exposure to SARS-CoV-2 vs those who were born during the same time frame but with no in utero exposure to SARS-CoV-2.

### Data Extraction

Data abstraction of included articles was performed by 2 independent authors (K.H. and N.Z.) using a standardized sheet. The following data were abstracted: author’s name, publication year, study design, the sample size of pandemic and prepandemic cohorts, maternal SARS-CoV-2 status during pregnancy, patients’ perinatal data, the definition of NDI, and age at neurodevelopmental screening. The corresponding author was contacted in case of missing data in an included study.

### Quality Assessment

Newcastle-Ottawa Scale (NOS) was used to evaluate the quality of included studies and the risk of bias. NOS comprises participant selection, comparability of study groups, and assessment of outcome or exposure. A score of 7 or above was considered to be of high quality.^18^ Quality assessment selection was performed by 2 independent authors (K.H. and N.Z.), and discrepancies were resolved by the third investigator (A.A.S.).

### Statistical Analysis

Statistical analysis was performed using Stata statistical software version 17 (StataCorp). Pooled effect sizes were presented as odds ratio (OR) with a 95% CI for categorical variables using DerSimonian and Laird method. Subgroup analysis was conducted to assess the risk of NDI according to the infantile age in which screening was performed (6 vs 12 months after birth). A 2-sided *I*^2^ test was used to examine heterogeneity across the included studies; *I*^2 ^≥ 50% and *P* < .05 indicate heterogeneity. A random-effects model was used due to the anticipated heterogeneity of included studies. Furthermore, publication bias was assessed using Egger and Begg tests.

## Results

### Demographic Characteristics

**Figure 2.**
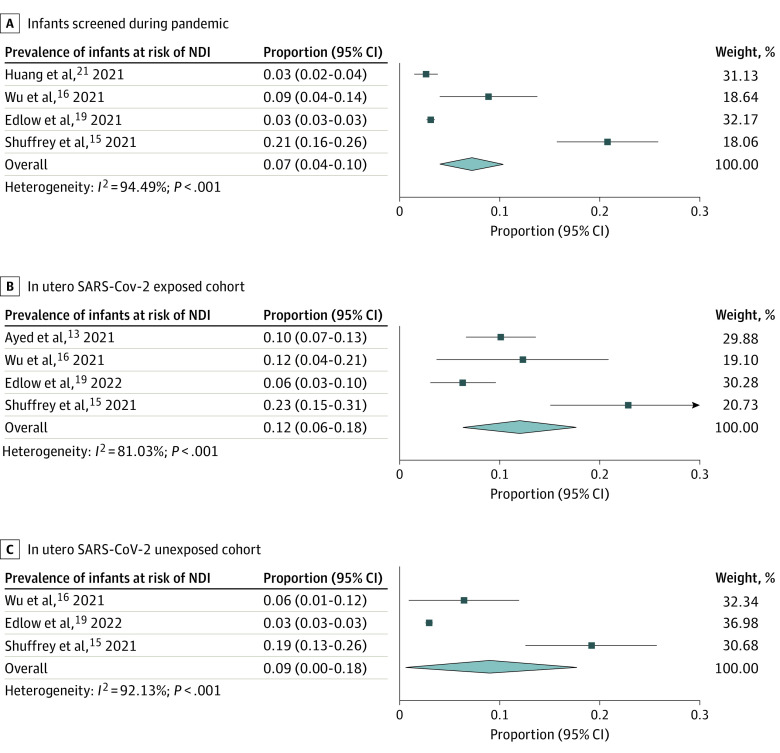
Prevalence of Risk for Neurodevelopmental Impairment (NDI) Among Infants Screened During the Pandemic, In Utero SARS-CoV-2–Exposed Cohort, and In Utero SARS-CoV-2–Unexposed Cohort Analyses used a random-effects DerSimonian Laird model.

The Table presents characteristics of the included studies, drawn from an initial search strategy inclusive of 1210 titles for screening. All included studies were published between 2021 and 2022. Three studies were conducted in the US,^15,19,20^ 3 in China,^16,21,22^ 1 in Kuwait,^13^ and 1 in Canada.^23^ Five studies were designed as prospective,^13,15,16,21,23^ 1 study as retrospective,^19^ and designs were not reported for 2 studies.^20,22^ A total of 21 419 infants who underwent neurodevelopmental screening were included in this systematic review, of whom 11 438 and 9981 were screened during the pandemic and prepandemic periods, respectively. Among those infants screened during the pandemic, 700 had documented confirmed maternal infection with SARS-CoV-2 during pregnancy, 7778 had no documented maternal infection, and the status of maternal SARS-CoV-2 exposure or infection was not known for the remainder (2960). As shown in Figure 2A, 7% (330 of 8992 infants; 95% CI, 4%-10%) of infants who had their neurodevelopmental screening during the pandemic were found to be at risk of NDI. Among the pandemic-born cohort, the prevalence of NDI among infants with confirmed maternal COVID-19 during pregnancy was 12% (77 of 691 infants; 95% CI, 6%-18%) (Figure 2B). For those who were born during the pandemic but had no maternal COVID-19 infection, the risk of NDI was 9% (330 of 8992 patients; 95% CI, 0%-18%) (Figure 2C).

**Table.** Characteristics of Studies Included in Both Quantitative and Qualitative Systematic Review and Meta-analyses

Source (Location)	Study design	Pandemic cohort, No. (No. COVID-19 positive/ No. COVID-19 negative)	Trimester of pregnancy at the time of COVID-19 infection	Prepandemic cohort, No.	Study period	Age at follow-up, mo	Infants, No./total No. (%)		NDI assessment tool	NOS score
							Incidence of NDI during pandemic	Incidence of NDI among COVID-19–positive population		
Studies included in systematic review and meta-analysis (quantitative analysis)										
Shuffrey et al,^15^ 2021, (US)	Prospective	255 (114/141)	30.7% in the third, 47.4% in the second, 21.9% in the first trimester	62	Oct 7, 2020, to Jun 17, 2021	6	53/255 (20.8)	26/114 (22.8)	ASQ-3	8
Wu et al,^16^ 2021, (China)	Prospective	135 (57/78)	93% in the third and 7% in the second trimester	NR	May 1 to Oct 31, 2020	3	12/135 (8.9)	7/57 (13.5)	ASQ-3	7
Imboden et al,^20^ 2021 (US)	NR	506 (NR)	NR	518	Oct 2020 to Jan 2021	6 and 12	NR	NR	ASQ-3	6
Huang et al,^21^ 2021 (China)	Prospective	831 (NR)	NR	5223	March 1 to May 15, 2020	6 and 12	24/830 (2.9)	NR	ASQ-3	7
Giesbrecht et al,^23^ 2022 (Canada)	Prospective	1623 (NR)	NR	4178	Started from Apr 2020	12	NR	NR	ASQ-3	6
Studies included in systematic review only (qualitative analysis)										
Cheng et al,^22^ 2021 (China)	NR	18 (9/9)	100% in the third trimester	NA	NR	3	NR	NR	ASQ-3	6
Ayed et al,^13^ 2021 (Kuwait)	Prospective	298 (298/0)	91.6% in the third, 6.7% in the second, and 1.7% in the first trimester	NA	Apr 1 to Dec 30, 2020	10-12	NR	30/298 (10.1)	ASQ-3	8
Edlow et al,^19^ 2021 (US)	Retrospective	7772 (222/7550)	72% in the third, 27% in the second, and 0.5% in the first trimester	NA	Between Mar and Sep 2020	12	241/7772 (3.1)	14/222 (6.3)	*ICD-10*	7

Abbreviations: ASQ-3, Age and Stage Questionnaire, Third Edition; *ICD-10*, *International Statistical Classification of Diseases and Related Health Problems, Tenth Revision*; NA, not applicable; NDI, neurodevelopmental impairment; NOS, Newcastle Ottawa Scale; NR, not reported.

### Neurodevelopment Screening During the COVID-19 Pandemic vs Before Pandemic

**Figure 3.**
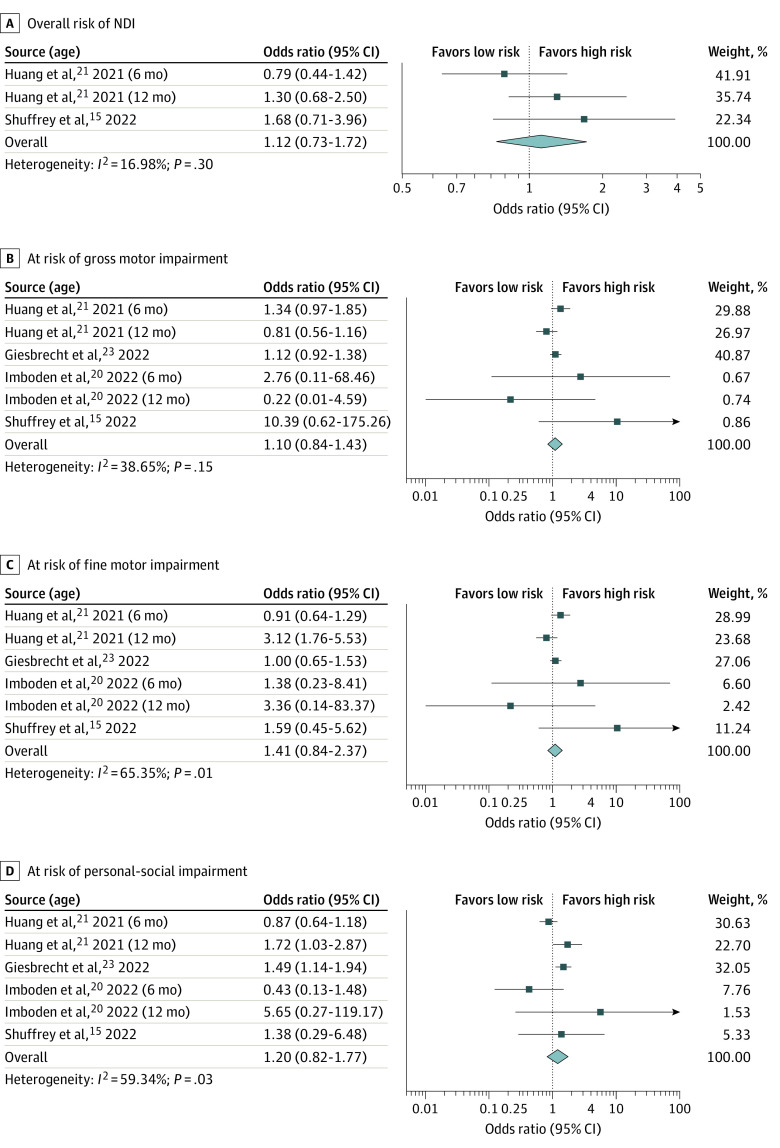
Meta-analysis of Overall Risk of Neurodevelopmental Impairment and Gross Motor, Fine Motor, and Personal-Social Domains Among Infants Screened During Pandemic vs Before Pandemic Analyses used a random-effects DerSimonian Laird model.

Overall, there was no significant difference regarding the overall risk of NDI, adjusted for prematurity, among infants screened during the pandemic compared with those screened before the pandemic (OR, 1.12; 95% CI, 0.73-1.72; *P* = .61; *I*^2^ = 17%). Additionally, there was no significant difference in terms of ASQ-3 domains, including gross motor (OR, 1.10; 95% CI, 0.84-1.43; *P* = .49; *I*^2^ = 39%), fine motor (OR, 1.41; 95% CI, 0.84-2.37; *P* = .20; *I*^2^ = 65%), personal-social (OR, 1.20; 95% CI, 0.82-1.77; *P* = .34; *I*^2^ = 59%), and problem-solving (OR, 0.97; 95% CI, 0.79-1.19; *P* = .75; *I*^2^ = 0.0%), except for an increased risk of communication impairment (OR, 1.70; 95% CI, 1.37-2.11; *P* < .001; *I*^2^ = 0.0%) among infants who were born during the pandemic (Figure 3 and eFigure 1 in the Supplement).

Subgroup analysis was performed according to the infantile age in which neurodevelopmental screening was performed. At age 6 months, the risk of NDI was comparable between pandemic and prepandemic screening groups. However, infants screened during the pandemic were more likely to have higher risk of communication (OR, 1.86; 95% CI, 1.15-3.00; *P* = .01; *I*^2^ = 70%) and personal-social (OR, 1.55; 95% CI, 1.22-1.96; *P* < .001; *I*^2^ = 0.0%) impairment at age 12 months (eTable 2 in the Supplement).

### Risk of NDI According to the Status of In Utero Exposure to SARS-CoV-2

**Figure 4.**
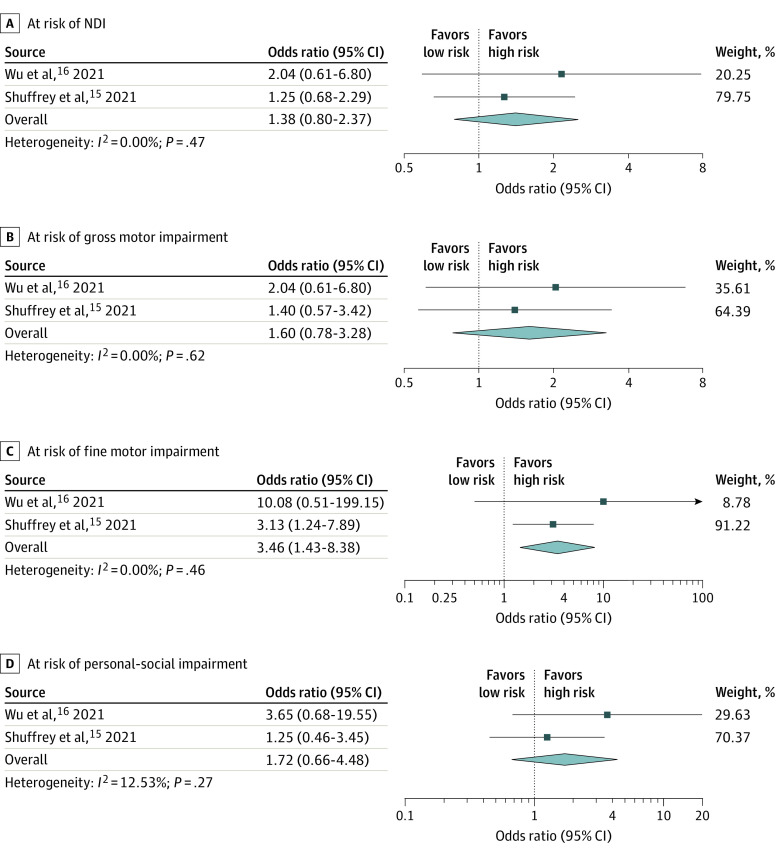
Meta-analysis of Overall Risk of Neurodevelopmental Impairment (NDI) and Gross Motor, Fine Motor, and Personal-Social Domains Among In Utero Exposed vs Unexposed Pandemic-Born Cohorts Analyses used a random-effects DerSimonian Laird model.

There was no significant difference regarding the overall NDI risk among infants born after gestational exposure to SARS-CoV-2 compared with those who had no exposure (OR, 1.38; 95% CI, 0.80-2.37; *P* = .24; *I*^2^ = 0.0%). Additionally, there was no significant difference in terms of ASQ-3 domains, including gross motor (OR, 1.60; 95% CI, 0.78-3.28; *P* = .46; *I*^2^ = 0.0%), communication (OR, 0.53; 95% CI, 0.15-1.89; *P* = .33; *I*^2^ = 0.0%), personal-social (OR, 1.72; 95% CI, 0.66-4.49; *P* = .27; *I*^2^ = 13%), and problem-solving (OR, 1.20; 95% CI, 0.57-2.51; *P* = .63; *I*^2^ = 0.0%), except for an increased risk of fine motor impairment (OR, 3.46; 95% CI, 1.43-8.38; *P* = .006; *I*^2^ = 0.0%) among infants who were exposed to SARS-CoV-2 in utero (Figure 4 and eFigure 2 in the Supplement).

### Quality Assessment

The overall score for the studies was between 6 and 8 out of a possible score of 9 (Table). Three studies^20,22,23^ had score of 6, 3 studies^16,19,21^ had score of 7, and 2 studies^13,15^ had score of 8. There was no publication bias for risk of overall NDI according to either the Egger test or the Begg test.

## Discussion

This systematic review and meta-analysis reports the potential association of the COVID-19 pandemic during pregnancy with neurodevelopmental outcomes during infancy. For infants screened before and during the pandemic, we found no significant differences in the overall risk of NDI. However, analyses of individual domains of development revealed infants screened during the pandemic were more likely to be at risk of communication impairment compared with their prepandemic counterparts. Our findings suggest that known maternal SARS-CoV-2 infection was associated with a significantly greater risk of fine motor impairment but no increased risk of impairment in any other domain of neurodevelopment.

Neurodevelopmental impairments presenting during infancy can be secondary to genetic susceptibility,^6^ preterm birth,^24^ perinatal inflammation, infection,^7^ diet,^25^ socioeconomic factors, maternal and placental health, and congenital anomalies. Maternal infection with SARS-CoV-2 during pregnancy is associated with preterm birth^5^ and inflammation^26^ and can lead to mental health problems including anxiety and depression.^27^ Unlike Zika virus, which has a fairly high maternal-fetal vertical transmission rate associated with a well-described spectrum of congenital manifestations, including microcephaly, cerebral palsy, and developmental delays,^11,28^ SARS-CoV-2 rarely crosses the placenta and is not associated with overt placental pathology.^14,29,30^ During the COVID-19 pandemic, financial strain, social isolation, and decreased family support have been associated with increased maternal depressive and anxiety symptoms in the perinatal period,^31^ which are known to be associated with neurodevelopmental and behavioral disorders. Higher levels of COVID-19–related stress were reported for both mothers and fathers of infants aged 0 to 6 months and were associated with insensitive parenting practices, including decreased emotional responsiveness in only mothers, which could lessen the reciprocal exchanges that support language development in early childhood.^32,33^ Additionally, opportunities to promote language and social development through new experiences outside the home, including visits with extended family and friends or attendance at a child care center, were lessened for many during the pandemic.

### Strengths and Limitations

 Among the most important strengths of this study included not limiting our included studies to only English publications, incorporating a highly sensitive search strategy inclusive of 1210 titles for screening, and a novel question with unanticipated findings. Our study is also prone to limitations in the heterogeneity of outcome measures, including limitations of quantitative analyses in 5 studies. An inherent limitation of the ASQ-3 questionnaire is its use as a screening instrument based on parent reports rather than objective assessment. Additionally, the sensitivity in detecting subtle but significant impairments in infancy is challenging compared with severe impairments or at later developmental stages. Given the novel finding of communication impairment as a result of being born during the COVID-19 pandemic, we were limited by a lack of data regarding the prevalence of social distancing among affected vs unaffected participants. Additionally, to better understand the subtle association of maternal SARS-CoV-2 infection during pregnancy with NDI warrants further investigation, with granular data on maternal-fetal COVID-19 illness and rigorous assessments of the potential for fetal SARS-CoV-2 exposures, which is beyond the scope of this review.

The ASQ-3 was administered by trained psychological evaluators in the Huang et al^21^ study, whereas in other included studies, the ASQ-3 is completed by caregivers or mothers. This might introduce variability in the results which should be interpreted with caution.

Clearly, although our observations raise potential concerns regarding the early developmental trajectory of children born during the COVID-19 pandemic, long-term follow-up behavioral assessments would be necessary to see whether this is borne out during early childhood or indeed whether catch-up occurring after the follow-up period is limited to the first year of life, and to extrapolate further into early childhood. As such, we consider the principal value of our current meta-analysis lies in its importance in generating novel hypotheses warranting further study.

## Conclusions

This systematic review and meta-analysis reveals a novel set of observations showing that being born and raised during the COVID-19 pandemic is associated with the risk of communication impairment among infants, with no evident association with other measures of neurodevelopment. More extensive studies with extended follow-up periods would provide more concrete insights into the long-term neurodevelopmental outcomes for infants and young children born during the pandemic and are, thus, warranted.

## Notes

Supplement. **eTable 1.** Search Strategy **eTable 2. **Subgroup Analysis of Neurodevelopmental Impairment According to the Age at Which Screening Has Been Performed **eFigure 1.** Meta-analysis Results on Risk of (a) Problem-solving and (b) Communication Among Infants Screened During Pandemic vs Before Pandemic **eFigure 2.** Meta-analysis Results on Risk of (a) Problem-solving and (b) Communication Among In Utero Exposed vs Unexposed Pandemic-Born Cohorts
