# Supplementary material for: COVID-19 Pandemic and Infant Neurodevelopmental Impairment: A Systematic Review and Meta-analysis
Source: JAMA Netw Open. 2022 Oct 28;5(10):e2238941. doi: 10.1001/jamanetworkopen.2022.38941 (PMC9617178; doi:10.1001/jamanetworkopen.2022.38941)
Supplement: Supplement. — eTable 1. Search Strategy eTable 2. Subgroup Analysis of Neurodevelopmental Impairment According to the Age at Which Screening Has Been Performed eFigure 1. Meta-analysis Results on Risk of (a) Problem-solving and (b) Communication Among Infants Screened During Pandemic vs Before Pandemic eFigure 2. Meta-analysis Results on Risk of (a) Problem-solving and (b) Communication Among In Utero Exposed vs Unexposed Pandemic-Born Cohorts [file jamanetwopen-e2238941-s001.pdf]

Supplemental Online Content

Hessami K, Norooznezhad AH, Monteiro S, et al. COVID-19 pandemic and infant neurodevelopmental impairment: a systematic review and meta-analysis. *JAMA Netw Open*. 2022;5(10):e2238941. doi:10.1001/jamanetworkopen.2022.38941

eTable 1. Search Strategy

eTable 2. Subgroup Analysis of Neurodevelopmental Impairment According to the Age at Which Screening Has Been Performed

eFigure 1. Meta-analysis Results on Risk of (a) Problem-solving and (b) Communication Among Infants Screened During Pandemic vs Before Pandemic

eFigure 2. Meta-analysis Results on Risk of (a) Problem-solving and (b) Communication Among In Utero Exposed vs Unexposed Pandemic-Born Cohorts

This supplemental material has been provided by the authors to give readers additional information about their work.

© 2022 Hessami K et al. *JAMA Network Open.*

e Table 1. Search Strategy

We searched PubMed, Web of Science, Embase for studies published in any language up to and including 25 March 2022 that reported the impact of COVID-19 pandemic on infantile neurodevelopment. We searched using keywords related to COVID-19 pandemic and neurodevelopment. Full search strategies for each database are provided below in a table.

Pubmed		
Number	Theme	Search query
1	COVID-19	("COVID-19" OR "Coronavirus" OR "Severe Acute Respiratory Syndrome" OR “Corona-virus" OR “2019nCoV” OR “Corona Virus” OR “COVID” OR “COVID19” OR “SARS CoV 2" OR “SARS-CoV” OR “SARS-CoV-2”)
2	Neurodevelopment	(“neurodevelopment” OR “neurodevelopmental” OR “development disorder*” OR “developmental disorder*” OR “Intellectual Disability” OR “Intellectual Developmental Disorder*” OR “mental retardation” OR “Global Developmental Delay” OR “Communication Disorders” OR “gross motor” OR “fine motor” OR “personal social” OR “problem solving”)
3	Search	#1 AND #2
Web of science		
1	COVID-19	("COVID-19" OR "Coronavirus" OR "Severe Acute Respiratory Syndrome" OR “SARS-CoV-2”)
2	Neurodevelopment	(“neurodevelopment” OR “neurodevelopmental” OR “development disorder*” OR “developmental disorder*” OR “Intellectual Disability” OR “Intellectual Developmental Disorder*” OR “mental retardation” OR “Global Developmental Delay”)
3	Search	#1 AND #2
Embase		
1	COVID-19	("COVID-19" OR "Coronavirus" OR "Severe Acute Respiratory Syndrome" OR “SARS-CoV-2”)
2	Neurodevelopment	(“neurodevelopment” OR “neurodevelopmental” OR “development disorder*” OR “developmental disorder*” OR “Intellectual Disability” OR “Intellectual Developmental Disorder*” OR “mental retardation” OR “Global Developmental Delay”)
3	Search	#1 AND #2

e Table 2. Subgroup Analysis of Neurodevelopmental Impairment According to the Age at Which Screening Has Been Performed

ASQ-3 domains	Age at screening	Number of studies	OR (95% CI), p-value	I^2, p-value
Gross motor	6 months	3	1.63 (0.73, 3.61), p=0.23	12%, p=0.32
	12 months	3	0.97 (0.71, 1.33), p=0.85	42%, p=0.18
Fine motor	6 months	3	0.96 (0.69, 1.33), p=0.79	0.0%, p=0.65
	12 months	3	1.83 (0.66, 5.08), p=0.25	80%, p=0.006
Problem solving	6 months	3	0.98 (0.63, 1.53), p=0.92	0.0%, p=0.53
	12 months	3	1.03 (0.66, 1.59), p=0.91	40%, p=0.19
Personal social	6 months	3	0.85 (0.64, 1.14), p=0.27	0.0%, p=0.46
	12 months	3	1.55 (1.22, 1.96), p<0.001	0.0%, p=0.62
Communication	6 months	3	1.69 (0.83, 3.44). p=0.15	0.0%, p=0.60
	12 months	3	1.86 (1.15, 3.00), p=0.01	70%, p=0.07

© 2022 Hessami K et al. *JAMA Network Open.*

e-Figure 1. Meta-Analysis Results on Risk of (a) Problem-Solving and (b) Communication Among Infants Screened During Pandemic vs Before Pandemic

(a)

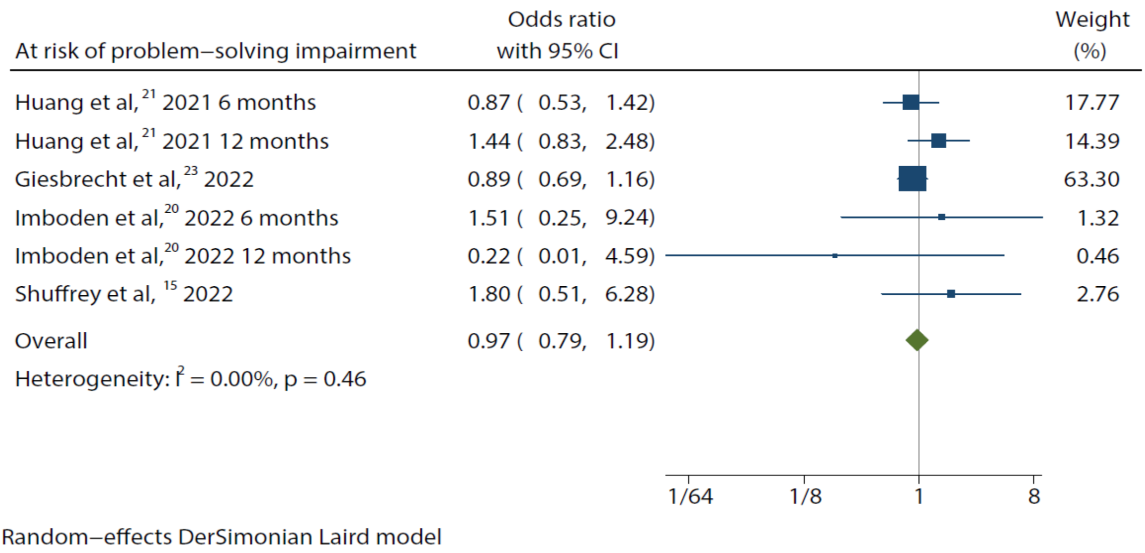

(b)

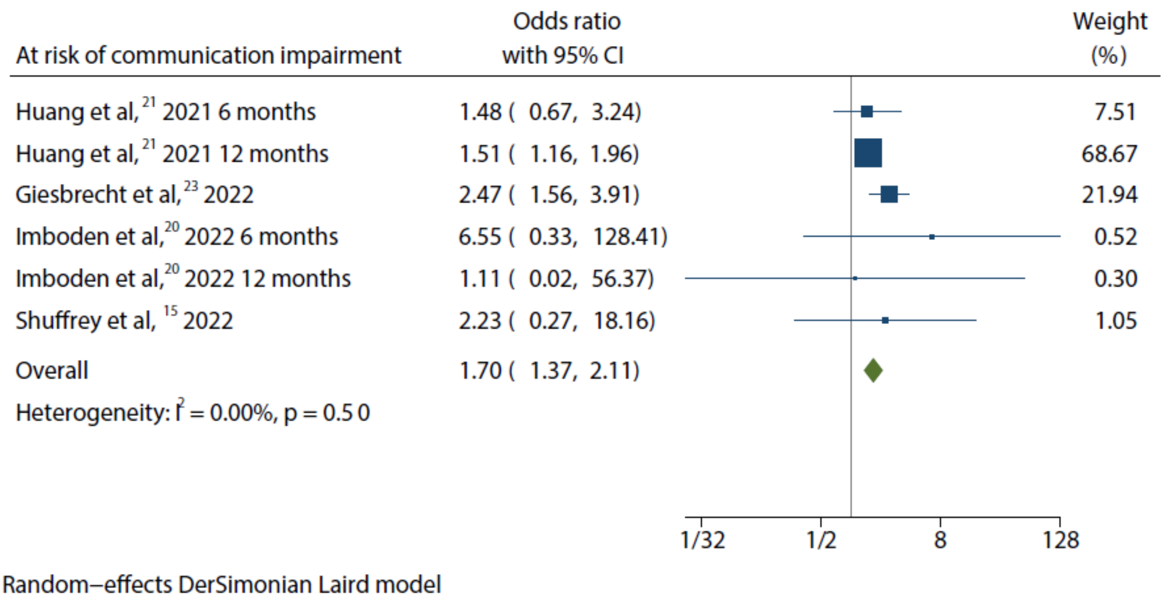

© 2022 Hessami K et al. *JAMA Network Open.*

e-Figure 2. Meta-Analysis Results on Risk of (a) Problem-Solving and (b) Communication Among In Utero Exposed vs Unexposed Pandemic-Born Cohorts

(a)

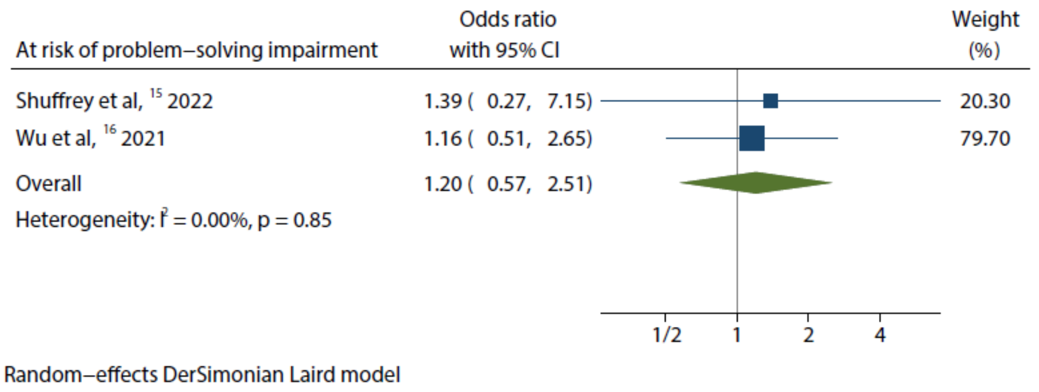

(b)

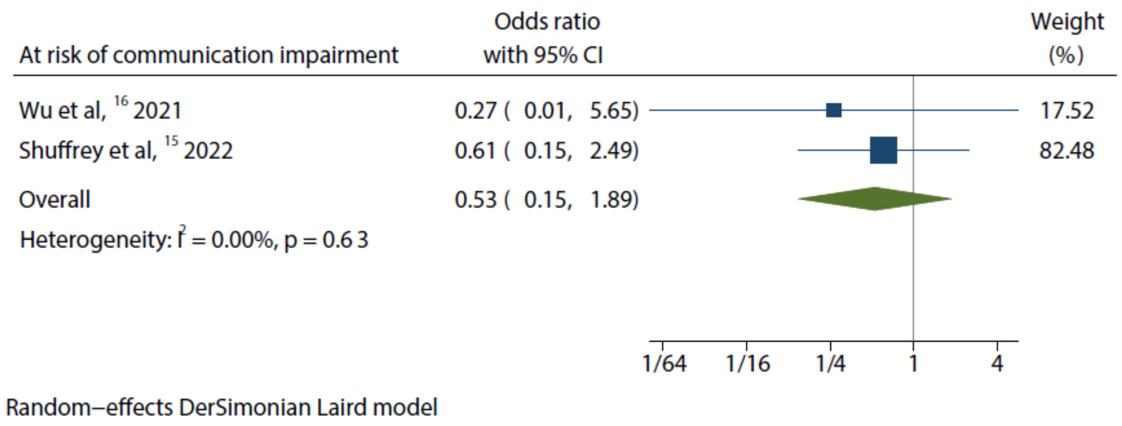

© 2022 Hessami K et al. *JAMA Network Open.*
